# Comparative energy value of discarded potato chips used as feedingredient in finishing diets for lambs

**DOI:** 10.5455/javar.2025.l1000

**Published:** 2025-12-30

**Authors:** Yesica Janeth Arteaga Wences, Jorge Luis Ramos Mendez, Alfredo Estrada Angulo, Jesús David Urías Estrada, Elizama Ponce Barraza, Lucía De Guadalupe Escobedo Gallegos, Daniel Alejandro Mendoza Cortez, Alberto Barreras, Luis Corona, Alejandro Plascencia

**Affiliations:** 1 Facultad de Medicina Veterinaria y Zootecnia, Universidad Autónoma de Sinaloa, Culiacán, Sinaloa 80260, México; 2 Instituto de Investigaciones en Ciencias Veterinarias, Universidad Autónoma de Baja California, Mexicali, Baja California 21100, México; 3 Facultad de Medicina Veterinaria y Zootecnia, Universidad Nacional Autónoma de México, Ciudad de México, México

**Keywords:** Carcass, Energy value, Finishing lambs, Performance, Potato by-product

## Abstract

**Objective:**

To determine the comparative energy value of discarded potato chips (DPC) used as a feed ingredient, the growth performance, carcass traits, and visceral mass were evaluated in 48 lambs.

**Materials and Methods:**

Lambs (29.89 ± 2.72 kg) were used in a randomized block design (8 replicates/treatment) in a 63-day feeding trial. DPC was composed mainly of malformed potato chips, which were removed before packaging. Its chemical composition was 96.92% dry matter, 7.11% CP, 39.65% ether extract, 2.84% neutral detergent fiber, and 46.03% starch. The DPC was included at 0%, 5%, and 10% in a total mixed ration (88:12 concentrate-to-forage ratio), replacing cracked corn grain.

**Results:**

Replacing corn with DPC did not affect dry matter intake (DMI), average daily gain, and gain-to-feed ratio. The observed dietary net energy (NE) was not modified by replacing corn with DPC and was close to (0.99) the NE expected according to the diet formulation. Using the replacement technique, the estimated NE for maintenance of DPC averaged 2.37 Mcal/kg. Kidney-pelvic-heart fat (KPH) and visceral fat were increased linearly (*p*= 0.04) as DPC increased in the diet, while the rest of the variables of carcass, shoulder tissue composition, and visceral mass were not affected.

**Conclusion:**

DPC included at up to a 10% level in the diet did not affect DMI, efficiency, and carcass traits but increased KPH and visceral fat. The average NE value of DPC is approximately 6.5% higher than that of cracked corn grain. However, due to its high fat content, careful consideration is necessary when formulating rations.

## Introduction

Finishing diets typically include large proportions of grains, such as corn, wheat, and barley, as energy sources, with corn being the most commonly used. Therefore, grain costs can significantly affect the overall cost of gain for finishing cattle. Therefore, unconventional feed resources and by-products are always sought to supplement or completely replace grains in finishing diets. Factors such as price, nutritional characteristics, and environmental pressures have led to the increasing inclusion of by-products in animal feed [1]. Due to its scale and economic significance, the potato industry is of great importance worldwide. Approximately 50% of production is processed into various products; within these, around 20% are destined for potato chips [2,3]. Potato chips are a popular snack worldwide. In Mexico, the potato used to manufacture potato chips for snacks is around 300,000 tons/year[4] . In preparation for the package, discarding the final product by not meeting quality standards can reach 1%–2%. Due to the chemical composition of potato chips, which contain around 45% starch and 30% fat[5,6] , discarded potato chips (DPC) are a potential feed for livestock. Considering that the metabolizable energy (ME) of starch is 3.85 Mcal/kg [7]and fat is 6.40 Mcal/kg[8] , potato chips potentially provide 3.65 Mcal ME/kg. This corresponds to 1.12-fold the ME of cracked corn grain (3.25 Mcal/kg)[9] . Therefore, from a nutritional perspective, DPC could replace corn grain in diets for growing, finishing broilers, pigs, and ruminants.

However, its high fat content (~35%) could limit its inclusion levels in growing-finishing diets for ruminants. This is because the energy value of supplemental fats is closely related to their fatty acid digestibility, and the fatty acid (FA) digestibility decreases (as well as their energy value) linearly as fat intake surpasses the ratio of 0.85 gm FA/kg body weight (BW) [10]. Furthermore, potato chips have a high content of salt (up to 3%), and salinity can reduce the acceptability of diets in ruminants; thus, a concern is whether the acceptability of diets containing DPC could negatively affect feed intake levels. The feeding value of several potato by-products (i.e., screen solids, potato peels, and potato frozen products, among others) is well documented[8,11,12]. Nevertheless, the information regarding the energy value of the product resulting from discarded potato chips as a feed ingredient for fattening ruminants is very limited. We hypothesized that DPC has, at least, a similar energy value to that of corn grain when supplemented at moderate levels (~10%) in finishing diets for lambs. For this reason, the objective of this trial was to determine the comparative energy value of discarded potato chips included at two levels in finishing diets for lambs. The variables evaluated were growth performance, dietary energetics, carcass traits, shoulder tissue composition, and visceral mass.

## Materials and Methods

### Ethical approval

All animal care and handling procedures were performed in accordance with the guidelines of the Animal Use and Care Committee of the Universidad Autónoma de Sinaloa (Protocol No. 0211024).

### Location of the study and ethical statement

The experiment was conducted at the Feedlot Lamb Research Unit of the Universidad Autónoma de Sinaloa in Culiacán, Mexico (24°46′13″ N, 107°21′14″ W). The study site has a subtropical climate and is situated 55 m above sea level.

### Animals, diet, and experimental design

As part of a study to evaluate the energy value of DPC in a finishing diet, as well as their effects on growth performance, dietary energy, carcass traits, and visceral organ mass, forty-eight intact male Pelibuey × Katahdin lambs (29.89 ± 2.72 kg) were used. The lambs were treated for parasites 3 weeks before beginning the experiment (Albendaphorte 10%, Animal Health and Welfare, Mexico City, Mexico), injected with 1,106 IU of vitamin A (Synt-ADE^®^, Fort Dodge, Animal Health, Mexico City, Mexico), and vaccinated against Mannheimia haemolytica (One shot Pfizer, Mexico City, Mexico). A total of eight weight groups were assigned to each of 24 pens, with two lambs per pen, and eight replicates per treatment. Lambs were weighed before the morning meal (electronic scale; TORREY TIL/S: 107 2691, TORREY Electronics Inc., Houston, TX, USA) and allocated to one of eight weight groups. Each pen has a surface area of 6 m² and includes overhead shade, buckets for watering, and 1 m fence-line feed bunks. During the 21 days preceding the experiment, lambs were adapted to the finishing diet and facilities by feeding ad libitum on a finishing diet without discarded potato chips (Table 1).

We obtained discarded potato chips from the manufacturer (Grupo Sabritas, PepsiCo de México, Culiacán, México). Table 2 provides information on the chemical characteristics of the DPC used and the corn grain that was replaced. Diets were supplied with DPC at levels of 0%, 5%, and 10%, and DPC was directly substituted for cracked corn grain (kg/kg) in diets. Mixed diets were prepared weekly using a paddle mixer (model 30910-7, Coyoacán, México) as follows: Step 1: We added grain to the mixer; Step 2: We added dry supplement (mineral-protein supplement) and soybean meal; Step 3: We allowed the feed to mix for at least 3 min; Step 4: after adding ground forage hay, DPC was added; [5] the mixture was then mixed for 7 min with molasses. As soon as the elaborate experimental diets had been mixed, they were stored in 120 kg capacity bags and carefully labelled in anticipation of their use. The nutritional composition and chemical characteristics of experimental diets are shown in Table 1. The animals were always provided with clean drinking water, as well as fresh feed twice a day at 0800 and 1400 h. Feed was provided in the morning (300 gm/lamb) and in the afternoon, with the amount adjusted daily to allow for a residual intake of 50 gm/kg daily. Each morning, residual feed was collected between 0740 and 0750 h. The amount of feed consumed was calculated by subtracting the quantity of feed refusals from the quantity offered. The weighing was conducted just prior to the morning feed on days 1, 35, and the final day (day 63). We adjusted gastrointestinal fill by multiplying LW by 0.96 and converting the initial and intermediate BW measurements into shrunk body weight (SBW). For the determination of final fasted body weight (FFBW), all lambs were fasted (from feed, but not from drinking water) for 18 h prior to being weighed individually.

**Table 1.** Composition of the experimental diets offered to lambs.

Item	Discarded potato chipslevel in diet (% DM)		
	0	5	10
Ingredient composition, %			
Sudan grass hay	12.00	12.00	12.00
Cracked corn^1^	62.00	57.00	52.00
Soybean meal	13.50	13.50	13.50
Discarded potato chips^2^	0.00	5.00	10.00
Molasses cane	10.50	10.50	10.50
Mineral-protein premix^3^	2.00	2.00	2.00
Chemical composition, % DM basis^4^			
Crude protein	14.42	14.35	14.10
Neutral detergent fiber	16.62	16.28	16.09
Starch	43.58	42.62	41.39
Ether extract	3.00	4.82	7.07
Ash	5.82	5.76	6.23
Net energy, Mcal/kg^4^			
Maintenance	1.94	1.94	1.94
Gain	1.30	1.30	1.30

1 Cracked corn grain was prepared by passing corn through rollers (46 × 61 cm, corrugated) that had been adjusted so that kernels were broken to obtain a final density of approximately 0.57 kg/l.

2 The chemical composition of discarded potato chips (DPC) was: Mineral-protein premix containing 57.5% CP, 21.8% Ca, 1.39% Mg, 15.0% NaCl, 0.015% Mn, 0.015% Se, 0.005% Co, and 0.100% Zn.

3 Dietary chemical composition was determined in our laboratory by analyzing subsamples collected and composited throughout the experiment.

4 Calculated based on the tabular net energy (NE) values for individual feed ingredients [NRC, 2007], except for DPC, because the net energy values for maintenance and gain of DPC for ruminants are unknown, to establish the observed to expected NE ratio, the NE assigned for DPC was considered equal to the replaced corn grain.

**Table 2.** Chemical composition was determined for the discarded potato chips and the replaced corn grain.

Item	Corn grain	Discarded potato chips
Dry matter content, %	88.16	96.92
Chemical composition, % DM basis		
Crude protein	8.65	7.11
Neutral detergent fiber	11.27	2.84
Starch	70.74	46.03
Ether extract	3.48	39.65
Ash	2.65	1.12
Bulk density, kg/l	0.566	0.438

#### Feed sample analysis

Each batch of feed was sampled. Feed refusals were collected daily and composited weekly for dry matter (DM) analysis (method 930.15) [13] to determine DM intake. Feed samples from the batch were subjected to the following analyses [13]: DM (oven drying at 105°C until no further weight loss, method 930.15), crude protein (CP; N × 6.25, method 984.13). Total starch was determined according to Zinn [14]. Neutral detergent fiber (NDF) was determined following procedures described by Van Soest et al. [15] (corrected for NDF-ash, incorporating heat-stable α-amylase using Ankom Technology, Macedon, NY, USA) and ether extract by the Soxhlet method.

#### Calculations of productive performance, dietary energetics, and the energy value of DPC

The initial SBW and the final fasted SBW recorded on day 63 were used to estimate average daily gain (ADG) and dietary net energy (NE). To calculate the average daily gain, the initial and final SBW were subtracted, and the result was divided by the total number of feeding days. Based on the average dry matter intake (DMI) observed throughout the 63-day experiment, feed efficiency was determined by dividing the ADG by the DMI. Observed-to-expected DMI and observed-to-expected dietary NE are two ways of evaluating the efficiency of dietary energy utilization in growth-performance trials. A projected energy intake is derived from estimates of diet NE concentration and measures of growth performance. This estimation of expected DMI is performed based on observed ADG, average SBW, and NE values of the formulated diet (1.94 and 1.30 Mcal for maintenance and gain, respectively: expected DMI, kg/day = (EM/1.94) + (EG/1.30), where EM (energy required for maintenance, Mcal/day) = 0.056 × SBW 0.75, EG (energy gain, Mcal/day) = 0.276 × ADG × SBW 0.75, and 1.94 and 1.30 are the NEm and NEg values contained in experimental diets which are calculated based on the ingredient composition [9]. For establishing the observed-to-expected NE ratio, the NE assigned to DPC was assumed to be equal to the replaced corn grain due to the lack of knowledge regarding the net energy values for maintenance and gain for ruminants. The coefficient (0.276) was taken from NRC [16], assuming a mature weight of 113 kg for Pelibuey × Katahdin male lambs. Based on the EM and EG values and the DMI observed during the experiment, the dietary net energy was calculated:


$$x\;=\frac{-b\pm \sqrt{b^{2}-4\mathrm{ac}}}{2c}$$

where x = observed dietary NE, Mcal/kg; a = −0.41 EM; b = 0.877 EM + 0.41 DMI +EG; and c = − 0.877 DMI [17].

The estimated net energy of DPC was calculated, given that the NEm value of DRC is 2.23 Mcal/kg [9], so the comparative NEm values for the DPC in each supplemented diet were estimated as follows:

For a 5% DPC supplementation level, NEm (Mcal/kg) of tested DPC = [NEm observed diet to 5% DPC—NEm observed diet to 0% 0%DPC)/0.05] + 2.23

For a 10% DPC supplementation level, NEm (Mcal/kg) of tested DPC = [NEm observed diet to 10% DPC—NEm observed diet to 0% 0%DPC)/0.10 + 2.23

The divisors 0.05 and 0.10 represent the amount of supplemental DPC in diets, and the 2.23 represents the NEm value of dry rolled corn NRC, 2007 [9] replaced by the DPC. The NE for gain (NEg) value of the tested DPC was derived from their estimated NEm values as follows: NEg, Mcal/kg = 0.877 NEm—0.41 [17].

#### Carcass characteristics, whole cuts, and tissue shoulder composition

On the same day, all lambs were slaughtered. After skinning the lambs, the gastrointestinal organs were separated and weighed, the abdominal and mesenteric fat was weighed, and the hot carcass weight (HCW) was determined. We obtained the following measurements after cooling carcasses (including kidneys and internal fat) in a cooler at 2°C to 1°C for 24 h: (1) fat thickness perpendicular to the longissimus thoracis (LM), measured between the 12th and 13th ribs of the ribeye; (2) LM surface area, calculated by using a grid reading of the cross section of the ribeye between the 12th and 13th ribs; and (3) kidney, pelvic, and heart fat. A kidney-pelvic-hearth (KPH) was manually removed from the carcass, weighed, and expressed as a percentage of the cold carcass weight (CCW). Dressing percentage is calculated by dividing HCW by FFBW. As per the guidelines set forth by the North American Meat Processors Association, each carcass was split in half. The left side was fabricated into wholesale cuts without trimming [18]. From the foresaddle, we obtained the rack, breast, shoulder, and foreshank, and from the hindsaddle, we extracted the loins, flank, and leg. A physical dissection of the shoulder was performed for the purpose of assessing the tissue composition of the shoulder.

#### Visceral mass data

There were several components of the gastrointestinal tract (GIT) that were removed and weighed, including the tongue, esophagus, stomach (rumen, reticulum, omasum, and abomasum), pancreas, liver, gallbladder, small intestine (duodenum, jejunum, and ileum), and large intestine (cecum, colon, and rectum). After washing, draining, and weighing the GIT, empty weights were obtained. It was calculated by subtracting the full digesta-free GIT from the SBW to determine the empty body weight (EBW). A fresh tissue basis was used to determine all tissue weights. Organ mass was expressed as grams of fresh tissue per kilogram of final EBW, where final EBW was defined as the final live body weight minus the total digesta weight. The total visceral mass was calculated by summing all visceral components (stomach complex, small intestine, large intestine, liver, lungs, and heart), including digesta. Calculation of the stomach complex included rumen, reticulum, omasum, and abomasum weights free of digesta.

#### Statistical analysis

Growth performance data (gain, gain efficiency, and dietary energetics), DM intake, and carcass data were analyzed as a randomized complete block design, with the pen as the experimental unit, using the MIXED procedures of SAS software 9.3 [19], with treatment and block as fixed effects and the experimental unit within treatment as a random effect. Whole cuts, tissue composition, and visceral organ mass data were analyzed using the MIXED procedures of SAS software [19], with treatment and pen as fixed effects, and the interaction between treatment and pen, as well as individual carcasses within pen by treatment, as random effects. Treatment means were separated using the “honestly significant difference test” (Tukey’s HSD test). In all cases, the least squares mean and standard error are reported, and contrasts are considered significant when the *p*-value is ≤ 0.05.

### Results and Discussion

There were no deaths or morbidities associated with this experiment, and no lambs had to be retired as a result. The average air temperature and relative humidity registered during the experiment were 26.3°C ± 3.8°C and 49.5% ± 2.9%, respectively. According to the air temperature and relative humidity that prevailed during the experiment, the temperature humidity index (THI) averaged 73.2 ± 5.1. Therefore, the ambient conditions were favorable to fattening the lambs [20].

Because DPC had a greater fat content and a lower content of CP and starch (Table 2), therefore, fat in diets that include DPC showed greater fat content and slightly lower CP and starch content. This was more evident as its participation in the diet increased (Table 1). The chemical characteristics of corn grain analyzed in the current study were very similar to those reported in previous studies [21,22] and in the current animal feed standards for ruminants[9] . There is very limited information available regarding the chemical composition of discarded potato chips. However, a chemical composition of 95.2% DM, 9.4% CP, 32.4% EE, 7% CF, and 3.9% ash was reported by Dhingra et al.[23] , which is similar to the chemical composition determined in the current study. In contrast, Sadq and Fatehi [3] reported similar starch content (44.8%), but lower fat (~22%) and higher NDF content (25.4%) in French-fried potatoes offered to the feedlot cattle. Potato tubers contain lower concentrations of fiber carbohydrates [24]. Processing potatoes into chips removes a significant portion of the fiber, and the frying process can further reduce the fiber content. Therefore, the lower fiber determined for DPC in the current experiment is expected. Due to the nature of waste and by-products in the industry, discrepancies in the chemical compositions of by-products are common among scientific reports. The type of substrate used (in this case, the specific potato variety and the agronomic conditions under which the potatoes were grown) and the processing and production methods affect the characteristics of the final product, as well as its waste and by-products. According to several studies, the average chemical composition of potato chips for marketing purposes was reported as 6.70% CP, 33.7% EE, 46.5% Starch, and 4.4% CF [5,6,25], which is in reasonable agreement with the chemical composition of the discarded potato chips used in the current study.

The effects of treatments on growth energetics and dietary energetics are shown in Table 3. Replacing corn with DPC did not affect dry matter intake. Based on DMI, lambs that received diets supplemented with DPC consumed 60 and 125 g of DPC for the 5% and 10% inclusion levels, respectively. Although several reports are available regarding the effects of potato waste (such as potato peels, screen solids, and frozen potato products) on the feed intake behavior of ruminants [12], the information on the effects of discarded potato chips on the intake and performance of lambs is very limited. Potato chips have a high content of salt (up to 3%) and fat (~20%–40%), both of which can reduce the acceptability of diets in ruminants; thus, a concern is whether the acceptability of diets containing DPC could negatively affect feed intake levels. In this sense, lactating Holstein cows that received a 35-day corn silage-based diet containing 10% French fry potato waste (~22% total fat), which partially replaced barley grain in the diet, showed a 4.3% reduction in DMI [3]. In nursery pigs, the inclusion of discarded potato chips in diets (DCP replaced corn in the basal diet) tended (*p* = 0.10) to reduce DMI when pigs were fed for a period of 14 days [26]. In the present experiment, inclusion of up to 10% of DPC in the diet did not affect DMI of the lambs. The basis of the effect of DPC in DMI can be affected by the level of inclusion, dietary composition, or the period of adaptation to DPC. When corn-silage-based diets are supplemented with oils or ingredients rich in oils, such as in the experiment performed by Sadq and Fatehi [3], the DMI of cows can be negatively affected [27]. On the other hand, in short periods of evaluation (i.e., 21 days or less), the palatability properties of ingredients (reflected as increases or decreases in dietary acceptability) can be more pronounced; in contrast, long-term periods allow gradual adaptation to the new ingredient, making those effects on consumption behavior less perceptible [28]. The current experiment lasted 63 days, which may have facilitated adaptation to DPC, thereby minimizing the potential effects of its palatability characteristics on lamb consumption.

**Table 3.** Effect of supplementation level of discarded potato chips (DPC) on growth performance, dietary energy, and estimated net energy value of DPC.

Item	Discarded potato chips level, % diet DM			SEM	*p*-value		
	0	5	10		Linear	Quadratic	
Days on test	63	63	63				
Pen replicates	8	8	8				
Live weight, kg^1^							
Initial	29.89	29.90	29.87	0.075	0.87	0.83	
Final	44.99	44.58	45.28	0.813	0.80	0.59	
Average daily gain, kg	0.240	0.233	0.245	0.013	0.81	0.58	
Dry matter intake, kg/day	1.238	1.202	1.247	0.043	0.89	0.46	
Gain to feed, kg/kg	0.194	0.194	0.196	0.005	0.71	0.86	
Observed dietary Net energy, Mcal/kg							
Maintenance	1.910	1.918	1.923	0.019	0.64	0.94	
Gain	1.264	1.272	1.276	0.017	0.64	0.94	
Observed to expected dietary Net energy ratio							
Maintenance	0.984	0.989	0.991	0.010	0.64	0.94	
Gain	0.973	0.979	0.982	0.013	0.64	0.94	
Observed to expected daily dry matter intake	1.024	1.019	1.016	0.012	0.64	0.94	
Estimated NE of DPC, Mcal/kg							
Maintenance		2.39	2.36				
Gain		1.69	1.66				

DM: dry matter.

1 Initial live weight (LW) was reduced by 4% to adjust for the gastrointestinal fill. Final LW was obtained following an 18–h fast without access to feed (access to drinking water was not restricted).

Lambs that received DPC in place of corn grain showed very similar DMI and ADG to lambs that received only corn grain in their diet (Table 3). Therefore, the gain-to-feed ratio was not different between treatments. This could be taken as an indicator that the energy values of DPC and corn grain are close. In nursery pigs, a slightly greater energy value for DPC was determined when corn grain was replaced in diets up to 20%. Although no statistical differences in weight gain were observed, a lower feed intake for pigs supplemented with DPC promoted an improvement in feed efficiency [29]. There is no information available regarding the net energy value of DPC, as determined by growth-performance response, for fattening ruminants. In dairy cows, replacing 10% of the barley grain with French fries’ waste improved the feed efficiency for lactation by 7.8% [3]. However, in that experiment, changes in both body weight and body condition score of cows were not measured; thus, the energy efficiency of DPC may have been overestimated. In an *in vivo* digestion trial using lambs [28], the ME of fried potato chips was estimated through apparent total tract digestion as 3.90 Mcal/kg (which was derived from total tract digestible energy multiplied by 0.93). That energy value is near the theoretical energy value if the chemical composition (fat and starch) contained in the fried potato chips used in that experiment is considered. The improvement in feed efficiency when cereal grains are replaced by fried potato products is attributed mainly to the greater energy contribution because of their higher fat content. However, ingredients that contain high amounts of fat and energy can be utilized less efficiently when their inclusion in diets surpasses certain levels. This is because the net energy value of supplemental fats is closely related to their FA digestibility, and the FA digestibility decreases (as well as their energy value) linearly as FA intake surpasses the ratio of 0.85 gm FA/kg BW [10]. In the current experiment, total fat intake was 1.55 and 2.34 fat intake/kg BW (approximately equivalent to 1.40 and 2.10 gm FA/kg BW) for 5% and 10% inclusion levels of DPC in diets, respectively; therefore, a decrease of 6% and 14% of the tabular net energy value for fat [9] is expected.

The observed dietary NE was not modified by replacing corn with DPC, averaging 1.916 ± 0.049 Mcal/kg, representing 0.99 of the net energy expected according to the diet formulation (Table 3). The observed diet energy density of a particular diet can be estimated based on DMI and growth parameters. This is a more effective method than the feed-to-gain ratio for assessing the efficiency of dietary energy utilization [30]. Additionally, this energetic approach allows us to compare the energy values of a known feed ingredient with those of a test feed ingredient by applying the replacement equation technique. In this sense, given that the net energy of maintenance value of the replaced corn grain is 2.24 Mcal/kg [9], thus applying the replacement technique (by comparing the observed NE of the test diets* vs.* the observed NE of the control diet), the NEm values for DPC were 2.39 and 2.36 Mcal/kg for 5% and 10% inclusion in diet, respectively. These values correspond to 7.1% and 5.8% above the energy of corn grain but 15% lower than expected based on the starch and EE content in the tested DPC. As mentioned earlier, the lower energy value observed here for DPC (in relation to the value expected by its chemical composition) can be explained, in part, by the high consumption of total lipids for the sheep that consumed the diets supplemented with DPC.

Hot carcass weights, dressing percentage, and LM area were not affected (*p* ≥ 0.61) by replacing corn with DPC. Similarly, carcass traits, shoulder tissue composition, and whole cuts were not affected by the inclusion of DPC in diets (Table 4). Generally, the slight reduction in starch content in diets by replacing cereal grain with other energy sources different from starch did not affect carcass characteristics and whole-cut yield. Carvalho et al. [31] replaced corn grain with crude glycerol at different levels in finishing diets for lambs. The reduction of up to 25% of total starch in diets by glycerin inclusion did not affect any carcass characteristics or percentage of whole cuts. In the current experiment, the reduction of starch was only 5% in the diet where corn was replaced by 10%.

Kidney-pelvic-hearth fat and visceral mass fat were linearly increased (*p* = 0.04) as DPC was increased in the diet, while the rest of the variables of carcass, shoulder tissue composition, whole cuts, and visceral mass (expressed as gm/kg empty body weight) were not affected by replacing corn with DPC (Tables 4,5). The addition of supplemental fat to diets can lead to an increase in fat carcass deposition, but it may not affect other carcass measurements or non-fat visceral mass to the same extent. In this way, replacing partially corn grain with feed ingredients rich in fat or with supplemental fats has a minor effect on non-fat carcass traits, tissue composition, and whole cuts [32,33]. Although increased dietary fat increases a certain proportion of carcass fat, it does not have this effect on the proportion of muscle fat, so tissue composition is generally unaffected [34]. In this way, the most consistent effect is the increase in back fat thickness (BFT) and KPH fat in feeding studies when the total fat in diets increases [35]. In feedlot lambs, however, this response has been less persistent, as some studies have reported that the total fat in finishing diets for lambs surpasses 7% resulting in increases in either BFT [36] , KPH [37], or both [38]. It may be due to a combination of the total fat level in the diet, degree of finish (a period when fats were supplemented), and weight at slaughter that causes these inconsistencies. Therefore, the total fat in the diet (up to 7%), the duration of fattening (63 days), and the slaughter weight (~45 kg) in the current study allowed us to detect differences in visceral fat deposition in lambs that received DPC.

**Table 4.** Effect of the supplementation level of discarded potato chips (FW) on carcass characteristics, shoulder tissue composition, and whole cuts of lambs.

Item	Discarded potato chips (% diet DM)			SEM	*p*-value	
	0	5	10		Linear	Quadratic
Hot carcass weight, kg	24.21	23.94	24.27	0.477	0.92	0.61
Dressing percentage	53.84	53.67	53.60	0.593	0.78	0.95
Cold carcass weight, kg	24.04	23.78	24.06	0.468	0.97	0.65
LM area, cm	18.20	18.32	18.60	0.436	0.99	0.61
Back fat thickness, cm	0.160	0.159	0.166	0.009	0.30	0.96
KPH, %	3.26	3.92	4.23	0.279	0.04	0.98
Shoulder composition, %						
Muscle	69.67	69.25	69.40	0.750	0.79	0.76
Fat	13.61	13.78	13.70	0.761	0.93	0.90
Muscle-to-fat ratio	5.27	5.08	5.23	0.403	0.94	0.74
Whole cuts, % of CCW						
Forequarter	47.91	47.73	48.14	0.331	0.63	0.47
Hindquarter	45.10	45.34	45.03	0.432	0.42	0.87
Neck IMPS205	10.30	10.52	10.20	0.356	0.84	0.55
ShoulderIMPS206	9.30	9.25	9.70	0.236	0.25	0.39
ShoulderIMPS207	16.87	17.08	16.59	0.185	0.31	0.15
Rack IMPS204	8.35	8.14	8.36	0.110	0.98	0.13
Breast IMPS209	5.09	4.99	5.41	0.118	0.08	0.09
RibsIMPS209A	8.20	8.03	8.46	0.125	0.17	0.08
Loin IMPS231	8.51	8.70	8.78	0.176	0.12	0.45
Flank IMPS232	7.39	7.16	7.52	0.208	0.67	0.27
LegIMPS233	29.58	29.51	29.30	0.282	0.35	0.60

LM:* m. longissimus thoracis*;KPH: kidney-pelvic-hearth fat; CCW= cold carcass weight.

**Table 5.** Effect of the supplementation level of discarded potato chips (DPC) on visceral mass characteristics of lambs.

Item	Discarded potato chips level (% diet DM)			SEM	*p* value	
	0	5	10		Linear	Quadratic
Full viscera (kg)	6.63	6.30	6.45	0.209	0.55	0.31
Dressing percentage	3.94	3.60	3.71	0.200	0.42	0.38
Empty body weight (%)	91.54	92.18	92.09	0.387	0.33	0.45
Organs, gm/kg empty body weight						
Stomach complex	24.69	24.47	24.19	0.435	0.43	0.95
Intestines	38.19	37.84	38.88	0.840	0.57	0.51
Hearth + lungs	22.30	21.95	22.68	0.671	0.70	0.52
Liver + spleen	18.07	17.80	18.76	0.376	0.23	0.20
Kidney	2.05	2.00	1.80	0.114	0.14	0.56
Omental fat	2.80	2.91	2.90	0.058	0.29	0.41
Mesenteric fat	22.74	25.48	26.28	1.133	0.05	0.66
Visceral fat	25.50	28.39	29.18	1.156	0.04	0.64

### Conclusion

Discarded potato chips are a feasible ingredient for fattening lambs. As a result of its chemical composition, it is an ingredient low in fiber (<3%), moderate in protein (7%), and high in energy, primarily due to its starch and fat content. The replacement of up to 10% of corn grain with DPC did not affect DMI, daily weight gain, feed efficiency, and the non-fat carcass traits, but increased back fat thickness and visceral fat mass. Using the replacement technique, the estimated NE value of DPC resulted in an average of 6.5% greater than the NE value of corn grain. However, due to its high fat and salt content, careful consideration is necessary when formulating rations.

### List of abbreviations

ADG, average daily gain; CCW, cold carcass weight; CP, crude protein; DMI, dry matter intake; DPC, discarded potato chips; FA/kg, fatty acids per kilogram; HCW, hot carcass weight; KPH, kidney-pelvic-heart fat; LM, longissimus thoracis; NDF, neutral detergent fiber; NE, net energy; NRC, National Research Council; SBW, shrunk body weight; Mcal/kg, megacalories per kilogram; kg, kilogram; ME/kg, metabolizable energy per kilogram; h, hour; gm, gram; gm/kg, grams per kilogram.
